# Molecular characteristics of CTX-M β-lactamase-producing and quinolone-resistant Escherichia coli among deer in a popular tourist spot in Japan

**DOI:** 10.1099/acmi.0.000882.v3

**Published:** 2024-11-12

**Authors:** Shiori Ikushima, Michiyo Sugiyama, Tetsuo Asai

**Affiliations:** 1 The United Graduate School of Veterinary Sciences, Gifu University, 1-1, Yanagito, Gifu, 501-1193, Japan; 2 Fukushima Regional Collaborative Center, National Institute for Environmental Studies, 10-2, Fukasaku, Miharu-machi, Tamura, Fukushima, 963-7700, Japan

**Keywords:** CTX-M β-lactamase, chromosomal insertion, *Escherichia coli*, quinolone-resistant, sika deer, WGS

## Abstract

**Introduction.** Antimicrobial resistance (AMR) is a growing global concern. Clonal lineages of CTX-M β-lactamase-producing *Escherichia coli* (CTXE) and quinolone-resistant *E. coli* (QREC) were disseminated among the deer population in a famous tourist destination (Nara Park; NP) in Japan.

**Hypothesis/gap statement.** The molecular characteristics of CTXE or QREC isolates, which could pose a threat to public health, have not been elucidated.

**Aim.** This study aimed to characterize the genetic traits of CTXE and QREC isolates derived from NP deer and compare them with lineages prevalent worldwide.

**Methodology.** Sixteen CTXE and three QREC isolates recovered from NP deer faeces between 2018 and 2020 were analysed using whole-genome sequencing (WGS). For endemic lineages, phylogenetic trees were constructed against the isolates registered in the EnteroBase database using the core genome SNP scheme.

**Results.** The most prevalent lineage in NP deer was ST3580. Several pandemic lineages, such as sequence type (ST) 38, ST58 and ST117, were included. The QREC lineages prevalent among deer were designated as extra-intestinal pathogenic *E. coli* or uropathogenic *E. coli* (UPEC). Thirteen of the 24 antimicrobial resistance genes (ARGs) were considered high-risk ARG families. Chromosomal integration of *bla*_CTX-M-15_ was observed in all plasmid-negative isolates. Phylogenetic analysis suggested relationships between NP isolates and isolates sourced from the environment or poultry.

**Conclusion.** ST3580 has a high potential for clonal dissemination. Furthermore, multiple clinically relevant lineages of CTXE and QREC are endemic in NP deer; however, they could be less virulent than isolates belonging to the same lineages, which could cause severe infectious diseases. Further studies are required to investigate the relationship between chromosomal integration of plasmid-encoded genes and the stable propagation of AMR bacteria in wildlife and the environment.

## Data Summary

Sequence data are available in the DNA Data Bank of Japan (https://ddbj.nig.ac.jp/search) under the accession numbers DRR538793 and DRR538795-DRR538811. All data are reported within the article and supplementary materials.

## Introduction

Antimicrobial resistance (AMR) is currently a major public health concern with growing severity. Multiple factors, such as overuse or misuse of antimicrobials, migration of humans and animals and inaccurate management of agricultural debris, promote the emergence and spread of AMR [1]. The One Health concept advocates the importance of an interdisciplinary approach to solving this problem. Wildlife, especially those thriving close to human settlements, are predisposed to acquire AMR bacteria (ARB) that are resistant to clinically relevant antimicrobials [2,3]. Once ARB colonize wildlife, they can be dispersed over long distances by animal migration [4] or disseminated via intra- and inter-species transmission [5,6].

Third-generation cephalosporins and quinolones are the highest priority critically important antimicrobials (HPCIA) according to the World Health Organization (WHO) [7] and are widely used in human and veterinary medicine. AMR to these antimicrobials makes the treatment of bacterial infections [represented by urinary tract infections (UTI) or bloodstream infections] challenging and increases the economic burden and risk of death [8]. AMR *E. coli* has been isolated from a wide range of environments, including wildlife [6,9, 10]. As most research in the prevalence of ARB in Japanese wildlife has targeted wildlife inhabiting mountainous areas [10], the genetic characteristics of CTX-M-producing or quinolone-resistant *E. coli* (QREC) in urban wildlife have rarely been reported [11].

Nara Park (NP), located in the urban area of Nara City, is a popular tourist destination in Japan. Wild sika deer (*Cervus nippon*) are densely populated on the plains of NP, and deer and humans (especially tourists) frequently come into contact with each other. Our previous study revealed that several lineages of CTX-M β-lactamase-producing *E. coli* (CTXE) and QREC isolates were widely distributed among NP deer [5,9]. Considering the close interaction between humans and deer, it is crucial to evaluate the pathogenicity of these isolates, as they could lead to public health concerns. Furthermore, the origin of the isolates (anthropogenic-associated lineages or wild lineages) provides fundamental knowledge on preventing the spread of ARB among deer and its transmission to humans.

Therefore, the aim of this study was to determine the molecular characteristics of CTXE and QREC isolates derived from NP deer, evaluate their impact on public health and explore the genetic relationship between NP isolates and lineages reported worldwide. Specifically, we performed whole-genome sequencing (WGS) on the representative isolates per pulsotype to detect antimicrobial resistance genes (ARGs), virulence genes (VGs), plasmid replicon type and other genotypic characteristics. We also evaluated the risk to humans by comparing genes or sequence types (STs) with those identified as human health risks. Furthermore, for endemic *E. coli* lineages derived from NP deer, phylogenetic trees were constructed and compared with a worldwide genome database.

## Methods

### Selection of isolates for WGS

For the 30 CTXE isolates derived from NP deer in 2018, we performed pulsed-field gel electrophoresis (PFGE) according to a previously described method [9] to select isolates for WGS, and four pulsotypes were obtained. For the 84 CTXE isolated from NP deer in 2019 and 2020, the PFGE analysis results have been previously reported, showing nine pulsotypes [9]. Because one of each pulsotype from the 2018 and 2019–2020 groups showed indistinguishable band patterns, we excluded a relevant pulsotype in the 2019–2020 group from the isolate selection for WGS analysis. Basically, one CTXE isolate per pulsotype (total of 12 pulsotypes) was analysed using WGS. If isolates belonging to the same pulsotype exhibited different AMR profiles, all isolates were examined. For QREC, we obtained 105 isolates, which were grouped into 15 pulsotypes in 2018 [5]. From these, we selected three pulsotype that were prevalent among deer, and one isolate per pulsotype was subjected to WGS analysis. In total, the numbers of isolates analysed for WGS were 16 and 3 in CTXE and QREC, respectively (Table 1). The numbers of sampled animals were 59 [5] and 144 [9] in 2018 and 2019–2020, respectively.

**Table 1.** The pulsotype and number of animals with each pulsotype, resistance profile, ST, serotype, *fimH* type, pathotype and chromosomal mutation of CTXE and QREC isolates derived from deer in NP from July 2018 to July 2020

			Previous study*			Present study				
Category	Isolate	Isolation month and year	Pulsotype†	No. of animals with each pulsotype	Resistance profile‡	ST	Serotype	FimH	Pathotype§	Chromosomal mutation||
CTXE	I159	September 2018	CA	2	A-CF-CT	58	H37	*fimH24*	–	–
	I69	July 2018	CB	1	A-CF-CT-G-K-T-N-CI	131¶	O25:H4¶	*fimH30*	ExPEC (UPEC)	*gyrA* (S83L, D87L)*parC* (S80I, E84V)*parE* (I529L)
	I52	July 2018	CC	26	A-CF-CT	3580	O8:H12	*fimH32*	–	–
	I197	September 2018	CC	26	A-CF-CT-N	3580	O8:H12	*fimH32*	–	–
	I242	September 2018	CD	1	A-CF-CT	38	O7:H15	*fimH24*	–	–
	C202	November 2019	CE	5	A-CF-CT-T	38	O1:H18	*fimH54*	–	–
	C203	November 19	CE	5	A-CF-CT-T-S	38	O1:H18	*fimH54*	–	–
	E247	July 2020	CE	5	A-CF-CT-T-CI-S	38	O1:H18	*fimH54*	–	–
	C172	November 2019	CE	5	A-CF-CT-T-CI	38	O1:H18	*fimH54*	–	–
	C209	November 2019	CF	1	A-CF-CT	2248	O78:H49	*fimH39*	–	
	C231	November 2019	CG	1	A-CF-CT	2280	O185:H16	*fimH87*	–	–
	E129	July 2020	CH	1	A-CF-CT-T-S	3580	O8:H12	*fimH32*	–	–
	C165	November 2019	CI	1	A-CF-CT	14 709	O86:H30	Not found	–	
	C201	November 2019	CJ	3	A-CF-CT	906	O150:H8	*fimH32*	–	–
	C180	November 2019	CK	1	A-CF-CT	155	H25	*fimH121*	–	–
	C197	November 2019	CL	1	A-CF-CT-K-T-N-CI-CH-S	46	O9 (O9a):H4	*fimH34*	–	*gyrA*(S83L)
QREC	I140	September 2018	QA	13	N	117	O119:H4	*fimH97*	UPEC	*gyrA* (S83L)*
	I218	September 2018	QB	10	N	515	O53:H38	Not found	–	*gyrA* (S83L)*
	I350	October 2018	QC	13	K-T-N-CI	162	O160(O8):H19	*fimH32*	ExPEC	*gyrA* (S83L, D87L)*parC* (S80I)*

* PFGE typing of CTXE isolated in 2019–2020 and QREC, antimicrobial susceptibility testing, and determining chromosomal mutation in QREC were conducted in our previous studies [5,9].

† Pulsotype was ordered based on the experimental data published onby Ikushima *et al*. [5] and Ikushima *et al*. [9].

‡ A, ampicillin; CF, cefazolin; CT, cefotaxime; G, gentamycin; K, kanamycin; T, tetracycline; N, nalidixic acid; CI, ciprofloxacin; CH, chloramphenicol; S, trimethoprim/sulfamethoxazole

§ ExPEC, extraintestinal pathogenic *E. coli*; UPEC, uropathogenic *E. coli.*

|| The designations present the substituted amino acids, and the position number (e.g. S83L) indicates the substitution of serine for leucine at amino acid 83 in the quinolone resistance-determining region. S, serine; L, leucine; D, aspartic acid; E, glutamic acid; V, valine; I, isoleucine.

¶ Previously reported in Sato *et al*. (2024) [12].

### WGS and bioinformatic analysis

Bacterial whole-cell DNA was extracted using a bead-bashing-based method (ZR BashingBead Lysis Tubes, ZYMO RESEARCH, Irvine, CA, USA) and purified using a QIAquick PCR Purification Kit (QIAGEN, Hilden, Germany). The DNA library was constructed using an Illumina DNA Prep (Illumina, Inc., San Diego, CA, USA), and 150 bp paired-end reads were sequenced on an Illumina iSeq sequencer, according to the manufacturer’s instructions (Illumina). Raw data were assembled *de novo* using CLC Genomic Workbench to generate contigs. The contig data were examined for acquired ARGs (ResFinder 4.1), VGs (VirulenceFinder 2.0), ST (MLST 2.0), serotype (SeroTypeFinder 2.0), *fimH* type (FimTyper 1.0) and plasmid replicon type (PlasmidFinder 2.1) using the Center for Genomic Epidemiology homepage (https://www.genomicepidemiology.org/). For I62, ST and serotype have been reported in our previous study [12], and these analyses were excluded. For C165, the ST was examined for re-designation by the EnteroBase module because allele *mdh* exhibited <100% identity in MLST 2.0 in the Center for Genomic Epidemiology database. VGs were categorized as virulence factors according to the criteria outlined in the VFDB database (http://www.mgc.ac.cn/VFs/main.htm). Bacteriocin-related genes were not distinctly categorized in VFDB; therefore, we categorized these genes as ‘bacteriocin’. To examine the pathotype, the isolates were checked to determine whether they met the criteria for the extraintestinal pathogenic *E. coli* (ExPEC) subpathotype established by Kubelová *et al*. [13]. No large plasmids were found in the isolates (I52, I197, E129 and C165); to elucidate the location of *bla*_CTX-M_ genes, the assembled draft genome of these isolates was annotated using DFAST [14] and aligned against the reference sequences of *E. coli* strain LH09-a chromosome (GenBank accession No. CP100544), *E. coli* strain EC0880B (GenBank accession No. OX460315) and *Klebsiella pneumoniae* strain F16KP0096 plasmid (GenBank accession No. CP052151) using Mauve software [15]. The location of *bla*_CTX-M_ was compared and visualized using genoPlotR [16].

The isolates assigned to ST3580 O8:H12 and ST117 O119:H4 were phylogenetically compared with the same ST and serotype isolates registered in the EnteroBase database (https://enterobase.warwick.ac.uk/species/index/ecoli) (57 isolates in ST3580 O8:H12 and 160 isolates in ST117 O119:H4). We selected these isolates because they were highly prevalent in the NP deer. Although E129 was assigned to ST3580 O8:H12 in MLST 2.0 offered by Center for Genomic Epidemiology, this isolate was excluded from phylogenetic analysis because EnteroBase assigned E129 to ST542. We firstly constructed a rapid neighbour-joining minimum spanning tree based on the core genome multi-locus sequence typing (cgMLST) V1+hierarchical clustering (HierCC) V1 scheme using GrapeTree [17] (Figs. S1 and S2, available in the online version of this article). GrapeTree clusters containing NP isolates were further compared by constructing a maximum likelihood tree based on the core genome SNP (cgSNP) scheme using RaxML [18]. Trees were rooted, and a minimum presence value of 95% was used. The ESC_LA3471AA and ESC_EA6264AA strains were used as reference genome of ST3580 O8:H12 and ST117 O9:H4, respectively. The assembled sequence data used for phylogenetic analysis were downloaded from the EnteroBase database; ARGs and VGs were analysed using NP deer isolate analysis methods, as described above.

## Results

The STs of CTXE were identified as follows: ST3580, ST38, ST906, ST58, ST155, ST2248, ST2280, ST46 and ST14709 (Table 1). The QREC was typed as ST117, ST162 and ST515. Five ST38 isolates consisted of two serotypes: O1:H18 and O7:H15.

**Fig. 1.**
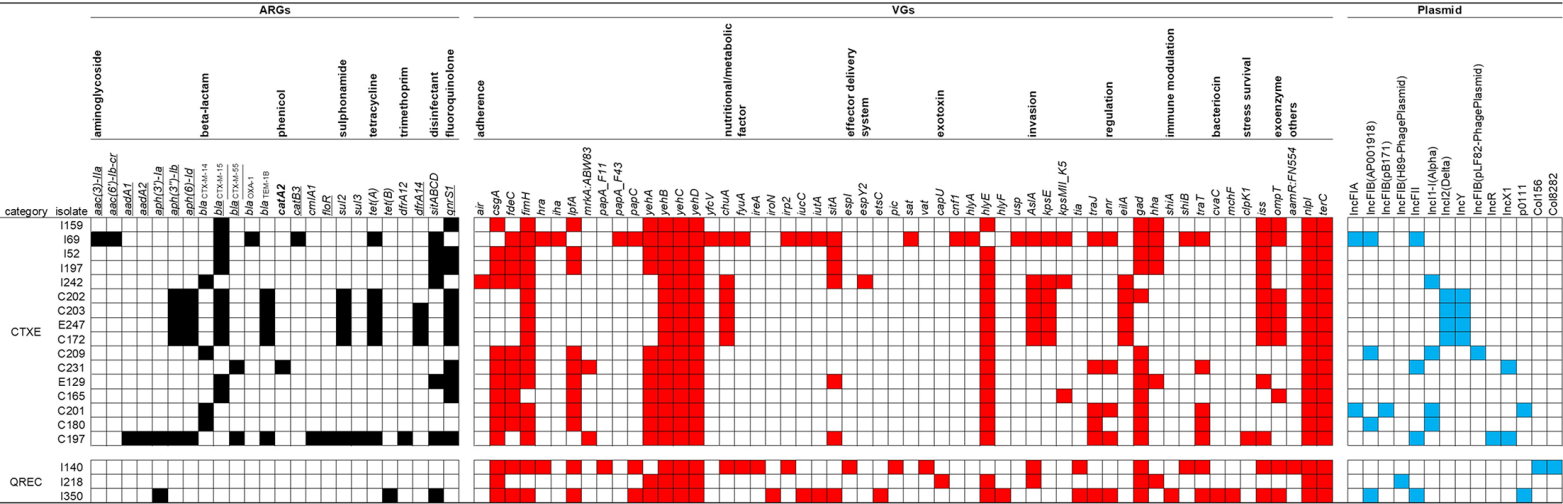
ARGs, VGs and plasmid replicon of CTXE and QREC isolates derived from deer in NP between July 2018 and July 2020. Black, red and blue cells represent the presence of each gene or plasmid replicon. Underlined and bolded ARGs are ranked as first and second high-risk ARGs, respectively [26].

The number of VGs in CTXE was the highest in strain I69 (ST131, 34 VGs) (Fig. 1). For QREC, I218, I140 and I350 encoded 12, 27 and 28 VGs, respectively. The VG profiles were identical within the pulsotypes, except for C202, which had an additional VG, *gad*. The VGs were categorized into 11 virulence factor groups based on their function: adherence (16 genes), nutritional/metabolic factor (eight genes), effector delivery system (six genes), exotoxin (six genes), invasion (five genes), regulation (four genes), immune modulation (three genes), bacteriocin (two genes), stress survival (two genes), exoenzyme (one gene) and others (three genes). I69 met the criteria of the ExPEC subpathotype of uropathogenic *E. coli* (UPEC). Being subject to the same criteria, I140 and I350 can be regarded as UPEC and ExPEC. Among the isolates, *fimH32* was prevalent across the three STs (ST3580, ST906 and ST162), followed by *fimH24* (I159 and I242; ST58 and ST38) and others.

A total of 24 ARGs were found: aminoglycoside (*aph(3'')-Ib*, five isolates; *aph(6)-Id*, five isolates; *aph(3')-Ia*, two isolates; *aac(3)-IIa*, one isolate; *aac(6')-Ib-cr*, one isolate; *aadA1*, one isolate and *aadA2*, one isolate), β-lactam (*bla*_CTX-M-15_, ten isolates; *bla*_TEM-1B_, five isolates; *bla*_CTX-M-14_, four isolates; *bla*_CTX-M-55_, two isolates and *bla*_OXA-1_, one isolate), phenicol (*catA2*, one isolate; *catB3*, one isolate; *cmlA1*, one isolate and *floR*, one isolate), sulphonamide (*sul2*, five isolates and *sul3*, one isolate), tetracycline (*tet(A*), six isolates and *tet(B*), one isolate), trimethoprim (*dfrA14*, three isolates and *dfrA12*, one isolate), disinfectant (*sitABCD*, seven isolates) and fluoroquinolone (*qnrS1*, 11 isolates) (Fig. 1). C197 acquired a broad spectrum of ARGs associated with eight categories of antimicrobials (15 ARGs).

**Fig. 2.**
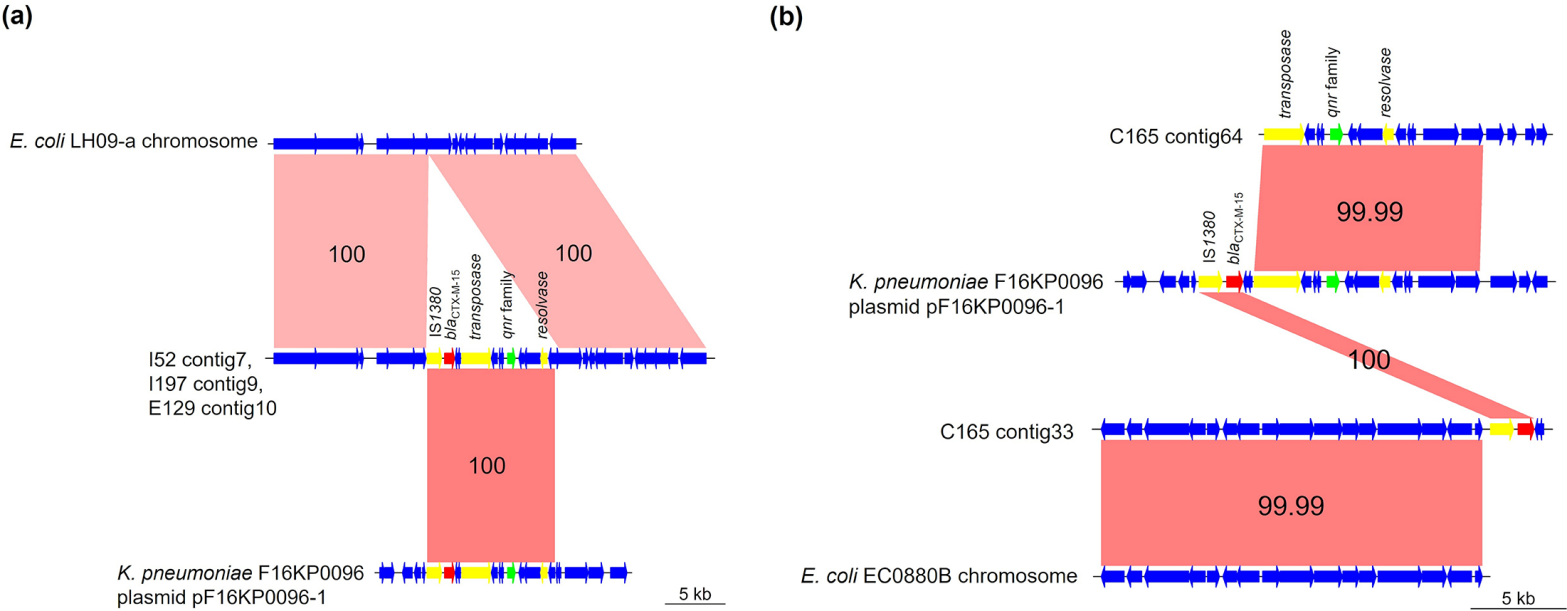
Chromosomal insertion of *bla*_CTX-M-15_ mediated by IS*1380*. (**a**) I52, I197 and E129 and (**b**) C165. In each isolate, the nucleotide sequence around *bla*_CTX-M-15_ was homologous with *Klebsiella pneumoniae* strain F16KP0096 plasmid (GenBank accession No. CP052151) ((**a**) I52 contig 7, I197 contig 9 and E129 contig10, between 167 085 and 178 237 bp; (**b**) C165 contig33, between 21 557 and 23950; C165 coting 64, between 106 and 12,105). Downstream and upstream of insertion exhibited high similarity with (**a**) *E. coli* strain LH09-a chromosome (GenBank accession No. CP100544) and (**b**) *E. coli* strain EC0880B chromosome (GenBank accession No. OX460315). Yellow, red, green and blue arrows represent genes encoding IS or transposase, β-lactamase, antimicrobial resistance other than β-lactamase and other functions including hypothetical protein, respectively. The numerical numbers in pink-shaded areas indicate the identity score (%) between two sequences.

The nucleotide sequence around *bla*_CTX-M-15_ showed high similarity to the reference sequences of the chromosome (I52, I197 and E129, *E. coli* strain LH09-a; C165, *E. coli* strain EC0880B) and the plasmids (all isolates, *K. pneumoniae* strain F16KP0096) (Fig. 2a, b). The simultaneous presence of IS, transposase and resolvase upstream and downstream of *bla*_CTX-M-15_ indicated that *bla*_CTX-M-15_ was transposed to the chromosome. All isolates that did not carry large plasmids had similar inserted components; the insertion was bound by the IS*1380* family at one end, which was located 1 518 bp upstream of *bla*_CTX-M-15_. The total length of the insertion was 11 152 bp in I52, I197 and E129 and, 15 406 bp in C165. I52, I197 and E129, which belonged to the same ST but showed different AMR profiles or pulsotype (Table 1), had the same genetic construction as the insertions.

**Fig. 3.**
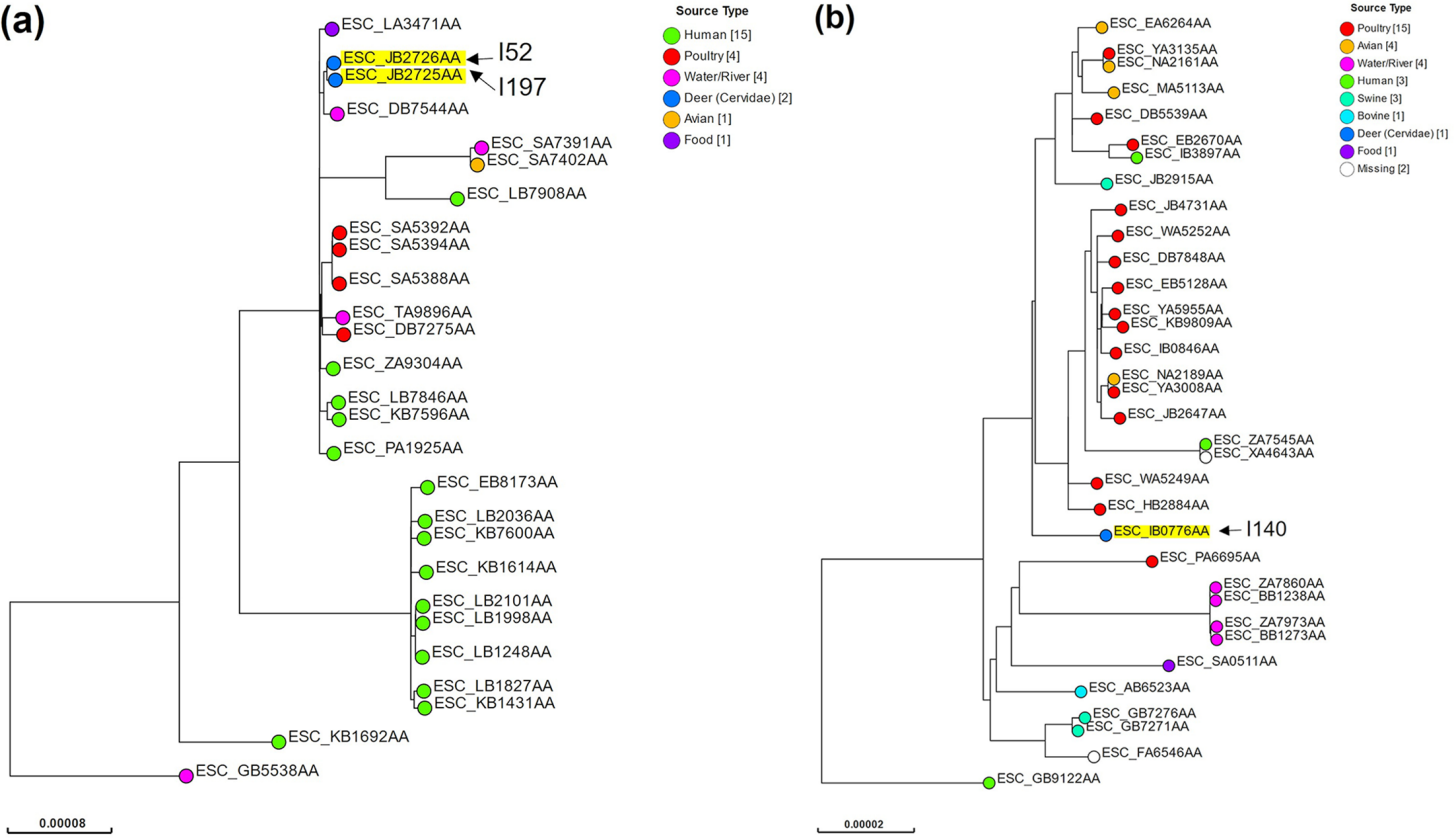
Maximum-likelihood tree based on cgSNP scheme compared with metadata from the EnteroBase database. (**a**) ST3580 O8:H12 and (**b**) ST117 O119:H4. GrapeTree clusters containing NP deer isolates were submitted for cgSNP analysis to construct a maximum likelihood tree based on RaxML (Figs S1 and S2, red circle). Each leaf was coloured according to source type and annotated using the EnteroBase sample barcode, which was highlighted in yellow in case of NP deer isolate.

cgSNP tree analysis revealed that I52 and I197 (ST3580 O8:H12) isolates belong to the clade composed of isolates sourced from poultry, human, water/river, avian and food (Fig. 3a). The ARG profile of ST3580 O8:H12 analysed in the study was characterized by *bla*_CTX-M-15_ and *qnrS1*, and NP isolates additionally encoded *sitABCD* (Table S1a). Notably, an isolate sourced from a wastewater treatment plant (WWTP) discharge in Otsu City, Japan, ~35 km from NP, was detected in the same clade (ESC_TA9896AA). However, the ARG profiles of NP isolates and ESC_TA9896AA were different: ESC_TA9896AA encoded *aadA1*, *bla*_OXA-10_, *qnrS1*, *cmlA1*, *floR*, *ARR-2*, *sul2*, *tet(A*) and *dfrA14*. The VG profiles were similar between the isolates compared in this study. I52 and I197 were the most closely related to an isolate sourced from water/river in China (ESC_DB7544AA). Regarding ST117 O119:H4, the sister clade of I140 was mainly composed of isolates sourced from poultry (Fig. 3b). Although 22 out of 33 ST117 O119:H4 isolates used for comparison in this study encoded ARGs, I140 did not carry any ARG (Table S1b). Furthermore, I140 lacked bacteriocin-associated genes or *hlyF*, which were present in the majority of ST117 O119:H4 isolates, from the EnteroBase database (30/33 and 29/33, respectively).

## Discussion

Our study results suggest that the most prevalent CTXE lineage in NP deer (ST3580) was not a pandemic lineage in humans. However, a recent study reported that ST3580 was the second most common ESBL lineage after ST131 in Malawi and Uganda and was disseminated in human, environmental and animal settings [19]. In this study, which was conducted in eastern Africa, ST3580 was found to be the most clonal among the five most prevalent lineages. Our study supports that ST3580 likely had a high potential for dissemination, irrespective of host species. Certain lineages detected in NP deer, such as ST38 and ST58, were relevant to human pandemic lineages [20]. ST38 is important as carbapenem-resistant *E. coli* (CREC), and 34.17% of CREC were typed as ST38 in Australia [21]. ST38 is also associated with UTI, accounting for 12% of UTI [20]. However, none of the ST38 isolates in the NP exhibited the traits of UPEC, enteroaggregative *E. coli* or carbapenem-resistant. Although ST58 was previously thought to be a cluster of environmental bacteria that belongs to phylogroup B1, most ExPEC strains are members of phylogroup B2 [20]; there is a growing concern regarding the increased responsibility of ST58 in bloodstream infections [22]. However, ST58 isolated from NP deer did not show any pathotype.

Regarding QREC, ST117 (I140) was spread among the NP deer and was positive in 13 out of 59 animals [5]. ST117 is a poultry-associated ExPEC lineage and the most prevalent ST in phylogroup G, which is uncommon but closely related to phylogroup B2 [23]. According to a study using sequence data of ST117 gathered globally, ST117 lineage carried multidrug resistance to HPCIA, and 93% of ST117 isolated from human was predicted to be originated from poultry, indicating that ST117 plays an important role as zoonotic ARB [24]. Furthermore, ST117 O119:H4 *fimH97* was shown to kill mice rapidly in mouse sepsis assays [23]. Although the ST117 QREC isolate obtained in this study was UPEC, it only showed resistance to nalidixic acid (NAL), encoded no ARG and carried fewer VGs than closely related ST117 isolates obtained from the public database (Table S1b). Another QREC lineage prevalent among NP deer, ST162 O160 (O8):H19 (I350), was also regarded as an ExPEC although ST162 is not a human ExPEC [25]. Overall, our data demonstrated the presence of some clinically relevant lineages in NP deer; however, they could be less virulent than the isolates belonging to the same lineage and causing severe infectious disease in humans or animals. Conversely, I62, expressed as ST131 [12], met the ExPEC (UPEC) pathotype criteria established by Kubelová *et al*. [13]. Furthermore, a previous study demonstrated that the I62 and human isolate belong to an identical cluster in a phylogenetic tree, implying dissemination between deer and humans [12]. The prevalence and genetic characteristics of ARB in NP deer need to be monitored.

Various ARGs were encoded by the CTXE and QREC isolates of the NP deer. Of these, 13 ARGs (five antimicrobial categories) were designated as high-risk ARG families [26] in the ARG risk ranking based on the WHO list [27]. The principal quinolone resistance mechanism of CTXE isolates in NP was an amino acid substitution in the quinolone resistance-determining region (QRDR). Consistent with a study showing that *qnrS1* confers ciprofloxacin (CIP) resistance but not necessarily NAL resistance [28], two *qnrS1*-positive isolates (E247 and C172) were resistant to CIP but susceptible to NAL [9]. In contrast, the isolates harbouring both *qnrS1* and QRDR mutations (I69 and C197) were resistant to both NAL and CIP [9].

We found that the most closely related isolates to I52 and I197 (ST3580 O8:H12) were sourced from water/river in Shandong, China (ESC_DB7544AA), in 2018, showing similar ARG and VG profiles. This indicated that there was an epidemiological link between NP deer and international sources. However, the nature of this dissemination, whether it was direct or indirect, remains unclear. I52 and I197 also showed phylogenetic similarity with the WWTP isolate obtained from the neighbouring city of NP (Otsu City, Shiga Prefecture) in 2018. NP I52 and I197 were isolated from deer faeces in July and September 2018, respectively, corresponding to the same time period of obtaining the Otsu isolate. The AMR in wastewater correlates with AMR in the human population, irrespective of the target bacterial species or antimicrobials [29]. However, ARG profiles were incompatible between NP isolates and Otsu isolates; therefore, we were unable to obtain solid evidence on local dissemination of this lineage. Consistent with a report by Cummins *et al*. [30], which demonstrated that ST117 is an APEC-associated lineage, we found that the sister clade of I140 was mainly composed of poultry- or avian-sourced isolates. All the isolates sourced from poultry or avian in the sister clade met the APEC VG criteria [13]; contrastingly, I140 did not. Our results indicated that I140 was phylogenetically close to APEC sourced from poultry or avian but could not determine the specific origin of the isolate.

We found the insertion of *bla*_CTX-M-15_ in all plasmid-lacking isolates. Insertions containing *qnrS1* as well as *bla*_CTX-M-15_ were translocated to the chromosome by the IS*1380* family, including IS*Ecp1*, which is responsible for the transposition of *bla*_CTX-M_ and other ARGs [31]. Chromosomal ARGs could be beneficial for the stable propagation of bacteria, regardless of the bacterial host’s habitat [32]. Regarding *E. coli* ST3580, the 11 kb chromosomal insertion of IS*Ecp1-bla*_CTX-M-15_-*qnrS1* was reported to exhibit a high growth rate [33]. In contrast, we found that the isolate belonging to the other lineage (ST14709) lacked plasmids and had insertions. Insertions of this isolate had similar genetic constructs as that of ST3580, matching well with the reference sequence of *K. pneumoniae* plasmid pF16KP0096-1. The reason for this coincidence remains unknown; however, IS*Ecp1* is strongly associated with several *bla*_CTX-M_ gene translocations [34]. A competitive assay is required to compare the growth rate of isolates to deepen our knowledge of the relationship between the chromosomal insertion of resistance genes and the survivability of CTXE isolates within the deer population.

Some common VGs among the isolates were related to adherence (*yehB*, *yehC* and *yehD*) and other functions (*NlPI* and *terC*). Yeh fimbriae, a product of the *yeh* gene, are highly conserved in *E. coli* and 92% of *E. coli* strains produce yeh [35]. *NlPI* plays a role in peptidoglycan multi-enzyme complexes by regulating and coordinating peptidoglycan activity and hydrolases [36]. Although *terC* is known to function in tellurite resistance, the *terC* operon is also involved in the resistance to infection, tolerance to oxidative stress, resistance to phagocytosis by macrophages, the ability of cells to adhere to epithelial cells and filamentous bacterial cellular morphology [37].

FimH is a fimbrial adhesin that functions in the adherence and infection of mammalian cells and biofilm formation [38]. FimH typing has shown high discriminative power in epidemiological studies and can sometimes be used alongside other typing methods [39]. Although it remains unknown whether specific *fimH* types are advantageous for stable proliferation in wildlife, *fimH32*, the most prevalent *fimH* type in NP deer, is the only common *fimH* type in CTXE isolates derived from sika deer culled in Gifu, Japan [10]. Accumulating knowledge about VGs encoded by AMR isolates recovered from wildlife will help us understand the properties of bacteria that contribute to the colonization of each host species and predict dissemination patterns within wildlife populations.

## supplementary material

10.1099/acmi.0.000882.v3 Uncited Supplementary Material 1.

10.1099/acmi.0.000882.v3 Uncited Supplementary Material 2.

## Abbreviations

AMR: antimicrobial resistance
ARGs: antimicrobial resistance genes
cgMLST: core genome multi-locus sequence typing
cgSNP: core genome SNP
CIP: ciprofloxacin
CREC: carbapenem-resistant *E. coli*
CTXE: CTX-M β-lactamase-producing *Escherichia coli*
ExPEC: extraintestinal pathogenic *E. coli*
HPCIA: highest priority critically important antimicrobials
NAL: nalidixic acid
NP: Nara Park
PFGE: pulsed-field gel electrophoresis
QRDR: quinolone resistance-determining region
QREC: quinolone-resistant *E. coli*
ST: sequence type
UPEC: uropathogenic *E. coli*
VGs: virulence genes
WGS: whole-genome sequencing
WHO: World Health Organization
WWTP: wastewater treatment plant

## References

[1] Velazquez-Meza ME, Galarde-López M, Carrillo-Quiróz B, Alpuche-Aranda CM (2022). Antimicrobial resistance: One Health approach. Vet World.

[2] Hassell JM, Ward MJ, Muloi D, Bettridge JM, Robinson TP (2019). Clinically relevant antimicrobial resistance at the wildlife-livestock-human interface in Nairobi: an epidemiological study. Lancet Planet Health.

[3] Wyrsch ER, Nesporova K, Tarabai H, Jamborova I, Bitar I (2022). Urban wildlife crisis: Australian silver gull is a bystander host to widespread clinical antibiotic resistance. mSystems.

[4] Ahlstrom CA, van Toor ML, Woksepp H, Chandler JC, Reed JA (2021). Evidence for continental-scale dispersal of antimicrobial resistant bacteria by landfill-foraging gulls. Sci Total Environ.

[5] Ikushima S, Torii H, Asano M, Suzuki M, Asai T (2021). Clonal spread of quinolone-resistant *Escherichia coli* among Sika Deer (*Cervus nippon*) inhabiting an Urban City Park in Japan. J Wildl Dis.

[6] Yossapol M, Yamamoto M, Sugiyama M, Odoi JO, Omatsu T (2021). Association between the blaCTX-M-14-harboring *Escherichia coli* isolated from weasels and domestic animals reared on a university campus. *Antibiotics*.

[7] World Health Organization (2024). WHO list of medically important antimicrobials: a risk management tool for mitigating antimicrobial resistance due to non-human use.

[8] Wozniak TM, Dyda A, Merlo G, Hall L (2022). Disease burden, associated mortality and economic impact of antimicrobial resistant infections in Australia. *Lancet Reg Health West Pac*.

[9] Ikushima S, Torii H, Sugiyama M, Asai T (2023). Characterization of quinolone-resistant and extended-spectrum β-lactamase-producing *Escherichia coli* derived from sika deer populations of the Nara Prefecture, Japan. J Vet Med Sci.

[10] Asai T, Usui M, Sugiyama M, Andoh M (2022). A survey of antimicrobial-resistant *Escherichia coli* prevalence in wild mammals in Japan using antimicrobial-containing media. J Vet Med Sci.

[11] Asai T, Sugiyama M, Omatsu T, Yoshikawa M, Minamoto T (2022). Isolation of extended-spectrum β-lactamase-producing *Escherichia coli* from Japanese red fox (*Vulpes vulpes japonica*). Microbiologyopen.

[12] Sato T, Uemura K, Yasuda M, Maeda A, Minamoto T (2024). Traces of pandemic fluoroquinolone-resistant *Escherichia coli* clone ST131 transmitted from human society to aquatic environments and wildlife in Japan. One Health.

[13] Kubelová M, Koláčková I, Gelbíčová T, Florianová M, Kalová A (2021). Virulence properties of mcr-1-positive *Escherichia coli* isolated from retail poultry meat. Microorganisms.

[14] Tanizawa Y, Fujisawa T, Nakamura Y (2018). DFAST: a flexible prokaryotic genome annotation pipeline for faster genome publication. Bioinformatics.

[15] Darling ACE, Mau B, Blattner FR, Perna NT (2004). Mauve: multiple alignment of conserved genomic sequence with rearrangements. Genome Res.

[16] Guy L, Kultima JR, Andersson SGE (2010). genoPlotR: comparative gene and genome visualization in R. Bioinformatics.

[17] Zhou Z, Alikhan N-F, Sergeant MJ, Luhmann N, Vaz C (2018). GrapeTree: visualization of core genomic relationships among 100,000 bacterial pathogens. Genome Res.

[18] Zhou Z, Alikhan NF, Mohamed K, Fan Y, Achtman M (2020). The enterobase user’s guide, with case studies on *Salmonella* transmissions, *Yersinia pestis* phylogeny, and *Escherichia* core genomic diversity. Genome Res.

[19] Musicha P, Beale MA, Cocker D, Oruru FA, Zuza A (2024). One Health in Eastern Africa: no barriers for ESBL producing *E. coli* transmission or independent antimicrobial resistance gene flow across ecological compartments. bioRxiv.

[20] Kocsis B, Gulyás D, Szabó D (2022). Emergence and dissemination of extraintestinal pathogenic high-risk international clones of *Escherichia coli*. Life.

[21] Huang J, Lv C, Li M, Rahman T, Chang Y-F (2024). Carbapenem-resistant *Escherichia coli* exhibit diverse spatiotemporal epidemiological characteristics across the globe. *Commun Biol*.

[22] Reid CJ, Cummins ML, Börjesson S, Brouwer MSM, Hasman H (2022). A role for ColV plasmids in the evolution of pathogenic *Escherichia coli* ST58. Nat Commun.

[23] Clermont O, Dixit OVA, Vangchhia B, Condamine B, Dion S (2019). Characterization and rapid identification of phylogroup G in *Escherichia coli*, a lineage with high virulence and antibiotic resistance potential. Environ Microbiol.

[24] Saidenberg ABS, Edslev SM, Hallstrøm S, Rasmussen A, Park DE (2024). *Escherichia coli* ST117: exploring the zoonotic hypothesis. Microbiol Spectr.

[25] Yasugi M, Hatoya S, Motooka D, Matsumoto Y, Shimamura S (2021). Whole-genome analyses of extended-spectrum or AmpC β-lactamase-producing *Escherichia coli* isolates from companion dogs in Japan. PLoS One.

[26] Zhang A-N, Gaston JM, Dai CL, Zhao S, Poyet M (2021). An omics-based framework for assessing the health risk of antimicrobial resistance genes. Nat Commun.

[27] World Health Organization (2019). 2019 antibacterial agents in clinical development an analysis of the antibacterial clinical development pipeline. https://iris.who.int/bitstream/handle/10665/330420/9789240000193-eng.pdf?sequence=1.

[28] Sumrall ET, Gallo EB, Aboderin AO, Lamikanra A, Okeke IN (2014). Dissemination of the transmissible quinolone-resistance gene qnrS1 by IncX plasmids in Nigeria. PLoS One.

[29] Chau KK, Barker L, Budgell EP, Vihta KD, Sims N (2022). Systematic review of wastewater surveillance of antimicrobial resistance in human populations. Environ Int.

[30] Cummins ML, Li D, Ahmad A, Bushell R, Noormohammadi AH (2023). Whole genome sequencing of avian pathogenic *Escherichia coli* causing bacterial chondronecrosis and osteomyelitis in Australian poultry. Microorganisms.

[31] Zong Z, Partridge SR, Iredell JR (2010). IS*Ecp*1-mediated transposition and homologous recombination can explain the context of *bla*_(CTX-M-62)_ linked to *qnrB2*. Antimicrob Agents Chemother.

[32] Yoon E-J, Gwon B, Liu C, Kim D, Won D (2020). Beneficial chromosomal integration of the genes for CTX-M extended-spectrum β-lactamase in *Klebsiella pneumoniae* for stable propagation. mSystems.

[33] Shawa M, Furuta Y, Mulenga G, Mubanga M, Mulenga E (2021). Novel chromosomal insertions of ISEcp1-bla_CTX-M-15_ and diverse antimicrobial resistance genes in Zambian clinical isolates of *Enterobacter cloacae* and *Escherichia coli*. Antimicrob Resist Infect Control.

[34] Tian SF, Chu YZ, Chen BY, Nian H, Shang H (2011). ISEcp1 element in association with bla(CTX-M) genes of *E. coli* that produce extended-spectrum β-lactamase among the elderly in community settings. Enferm Infecc Microbiol Clin.

[35] Wurpel DJ, Beatson SA, Totsika M, Petty NK, Schembri MA (2013). Chaperone-usher fimbriae of *Escherichia coli*. PLoS One.

[36] Banzhaf M, Yau HC, Verheul J, Lodge A, Kritikos G (2020). Outer membrane lipoprotein NlpI scaffolds peptidoglycan hydrolases within multi-enzyme complexes in *Escherichia coli*. EMBO J.

[37] Nguyen TTH, Kikuchi T, Tokunaga T, Iyoda S, Iguchi A (2021). Diversity of the tellurite resistance gene operon in *Escherichia coli*. Front Microbiol.

[38] Yoshida M, Kawamoto S, Kaneko S (2009). Effect of sugars on biofilm formation by *Escherichia coli* O157:H7 (in Japanese with English abstract). J Appl Glycosci.

[39] Neamati F, Moniri R, Khorshidi A, Saffari M (2020). Structural and functional characterization of the FimH adhesin of uropathogenic *Escherichia coli* and its novel applications. J Bacteriol Mycol.

