# Supplementary material for: Molecular characteristics of CTX-M β-lactamase-producing and quinolone-resistant Escherichia coli among deer in a popular tourist spot in Japan
Source: Access Microbiol. 2024 Nov 12;6(11):000882.v3. doi: 10.1099/acmi.0.000882.v3 (PMC11556432; doi:10.1099/acmi.0.000882.v3)
Supplement: Uncited Supplementary Material 1. [file acmi-6-00882-s001.pdf]

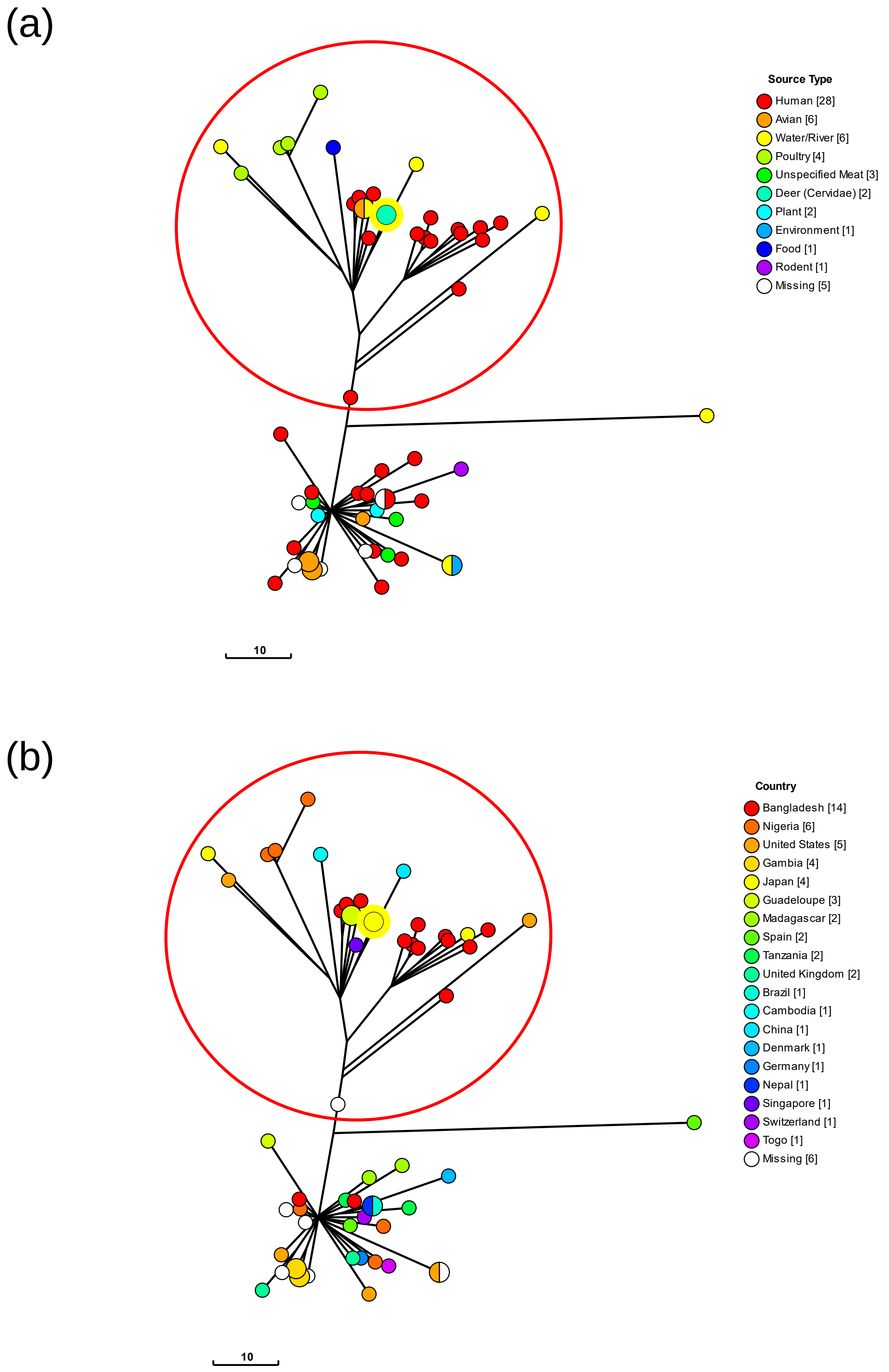

Supplementary Figure 1. Phylogenetic analyses of ST3580 O8:H12 isolates derived from Nara Park deer (circled in yellow) compared with sequence data from the EnteroBase database. Rapid neighbor-joining minimum spanning trees (GrapeTree) were constructed based on core genome multilocus sequence typing (cgMLST) V1 + hierarchical clustering (HierCC) V1. Each node was colored according to the (a) source type and (b) country. Isolates circled in red were further examined for core genome single nucleotide polymorphism (cgSNP) analysis.

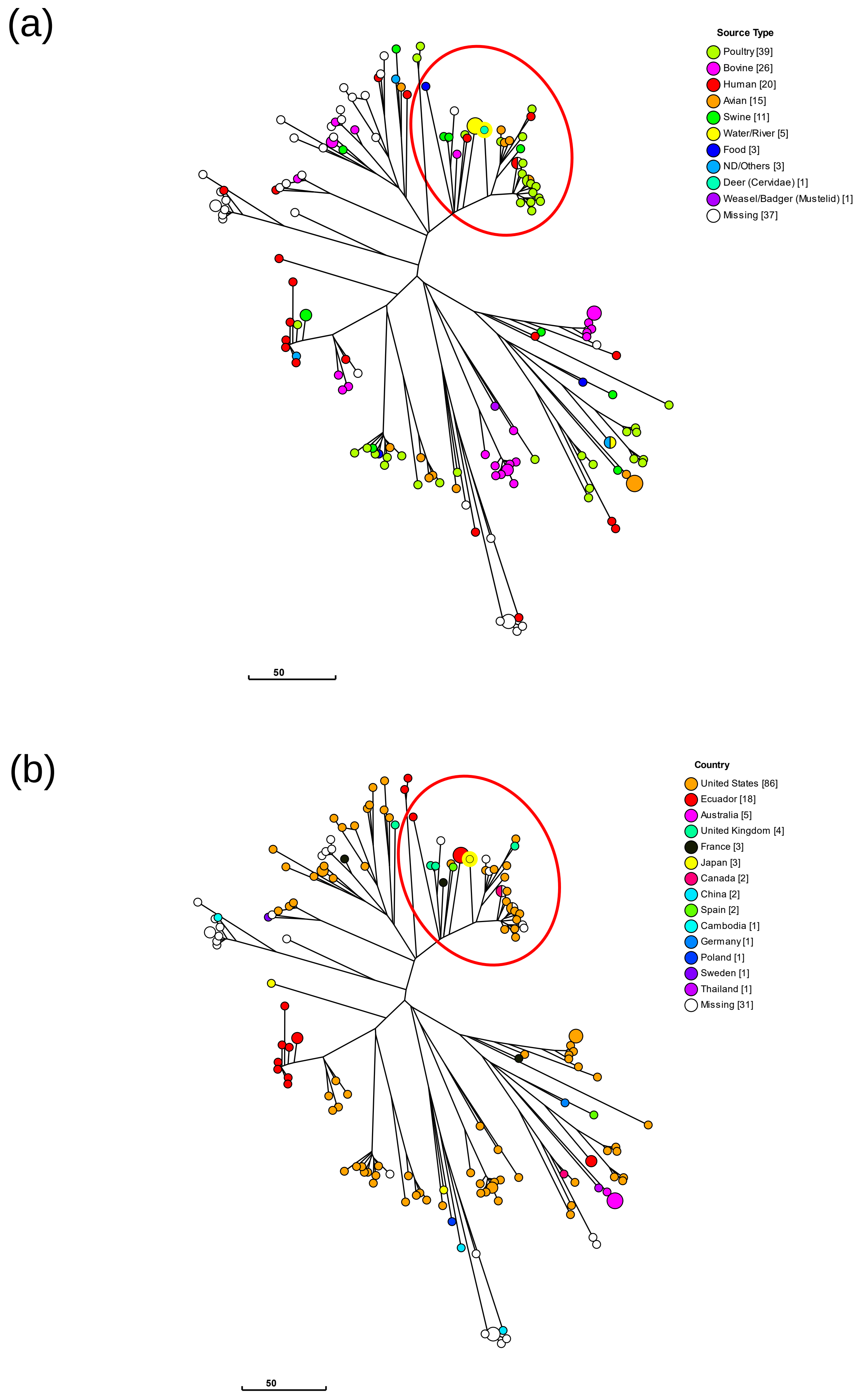

Supplementary Figure 2. Phylogenetic analyses of ST117 O119:H4 isolate derived from Nara Park deer (circled in yellow) compared with sequence data from the EnteroBase database. Rapid neighbor-joining minimum spanning trees (GrapeTree) were constructed based on core genome multilocus sequence typing (cgMLST) V1 + hierarchical clustering (HierCC) V1. Each node was colored according to the (a) source type and (b) country. Isolates circled in red were further examined for core genome single nucleotide polymorphism (cgSNP) analysis.
